# Blastic plasmacytoid dendritic cell neoplasm: A single centre Indian experience

**DOI:** 10.1016/j.lrr.2025.100560

**Published:** 2025-12-26

**Authors:** Paramasivam Sabitha, Aastha Goel, Santhosh Kumar, Chethan R, Sanal Fernandes, Nitin Jain, Naveen Pemmaraju, Atul Sharma, Ranjit Kumar Sahoo

**Affiliations:** a Department of Medical Oncology, Dr. B. R. Ambedkar IRCH, All India Institute of Medical Sciences, New Delhi, 110029, India; b Department of Medical Oncology, Manipal hospitals, Mangaluru, 575001, India; c Department of Leukemia, The University of Texas MD Anderson Cancer Center, Houston, TX, USA; d Medical Oncology Department, Max Hospital, Saket, New Delhi, 110017, India

## Abstract

BPDCN is an uncommon haematological malignancy recognised as a distinct entity by the WHO in 2016. It typically presents with skin lesions and involves bone marrow, lymph nodes, and CNS, affecting all age groups. The disease poses significant diagnostic challenges, with limited data on optimal treatment strategies, further contributing to its dismal prognosis. Standard regimens include ALL-like or AML-like therapies, particularly in older adults. We outline our experiences managing three consecutive BPDCN patients, emphasising the unique challenges oncologists face when treating this malignancy. This underscores scarcity of effective therapies, limited expertise, and intricacies involved in managing such a complex disease.

## 1 Introduction

BPDCN, a rare and distinct malignancy derived from dendritic cell lineage, markedly different and more elusive than other myeloid malignancies [1]. This clinically aggressive entity has been previously referred to by various nomenclatures, including CD4+/CD56+ hematodermic malignancy, agranular CD4+ natural killer (NK) cell leukaemia, and CD56+/TdT+ blastic NK cell tumor. Aberrant transformation of plasmacytoid dendritic cells (pDCs) precursors is a key event, characterized by expression of markers such as CD4, CD56, CD123, and TCL-1. TCF4, an E-box transcription factor, plays crucial regulatory role in the development of pDCs [2,3]. This unique malignancy predominantly involves skin, with or without involvement of the bone marrow, lymph nodes, or CNS. The overall incidence is estimated to be 0.04/100,000 population. While the median age at diagnosis is 65 years, BPDCN has been reported across all age groups, including children, adolescents, and young adults. The disease primarily affects men. It is associated with significantly poor prognosis, with a median overall survival ranging from 9-23 months. Due to its rarity and diagnostic complexities, achieving treatment consensus remains highly challenging [4]. Current treatment modalities primarily include AML-like regimens (e.g., 3+7 regimen, azacitidine and venetoclax) and ALL-like regimens (e.g., hyper-CVAD—cyclophosphamide, vincristine, doxorubicin, and dexamethasone; BFM chemotherapy), mainly utilized in young, fit adults. These regimens often followed by hematopoietic stem cell transplantation (HSCT) during the first complete remission (CR) [5,6]. For elderly and unfit patients, where cytotoxic regimens pose significant challenges, azacitidine and venetoclax (A+V) have shown promising outcomes [7]. However, CR rates remain suboptimal at 40–60%, largely due to high relapse rates and short durations of response, which often hinder the ability to bridge patients to HSCT [8]. To date, there are no prospective trials or established treatment guidelines for the use of these induction regimens in BPDCN. The most notable advancement in treatment has been the development of Tagraxofusp (TAG), a CD-123-targeted immunotoxin, which has demonstrated an overall response rate (ORR) of 90% in frontline settings, with 45% of patients successfully bridged to HSCT [9].

We report three BPDCN cases treated at our centre, highlighting clinical features, diagnostic workup, and therapeutic challenges in both frontline and salvage settings.

## 2 Case details

### 2.1 Patient case #1

A 58-year-old man presented with multiple large erythematous papulonodular skin lesions distributed across neck, face, trunk, and bilateral upper and lower limbs. Laboratory investigations revealed a WBC count of 2,180 cells/microliter. A skin biopsy demonstrated diffuse dermal infiltration by intermediate-sized cells immuno-positive for CD4, CD56, and CD123, consistent with BPDCN. There was no evidence of CNS or bone marrow (BM) involvement. BM aspiration showed 8–10% plasma cells, a serum M protein level of 1.1 g/dL and no myeloma-defining events, suggestive of monoclonal gammopathy of undetermined significance (MGUS). Whole-body (WB) PET-CT scan revealed FDG-avid multiple cutaneous thickenings and left intra-parotid lymph node. He was initiated on Hoelzer protocol (ALL-like regimen), achieving clinical benefit by improvement in skin lesions, during induction; however, he subsequently experienced cutaneous progression. Treatment was switched to A+V. After two cycles, skin lesions worsened and subsequently treated with multiple therapies, including three cycles of decitabine with venetoclax (D+V), three cycles of CLV (cladribine, low dose cytarabine and venetoclax), three cycles of VRD (bortezomib, lenalidomide and dexamethasone), four cycles of mini-CHOP (cyclophosphamide, vincristine, doxorubicin, prednisolone) and three cycles of Gemox (gemcitabine and oxaliplatin). He was deemed ineligible for allogeneic hematopoietic stem cell transplantation (Allo-HSCT). Following three cycles of Gemox, he is in stable disease and currently on follow-up 7 months (Table 1).

**Table 1:** Clinical features and treatment details.

Case	Age (yrs)	Gender	System involved	Treatment summary	Disease response	Status last follow up
Patient 1	58	male	Isolated skin	Hoelzer protocol, A+V, D+V, CLV, VRD, miniCHOP, Gemox	SD	Alive
Patient 2	16	female	Isolated left breast skin	Hoelzer protocol, hyperCVAD, allo-HSCT	CR	Alive
Patient 3	41	male	Bone marrow	A+V, ICiCLe-ALL protocol, allo-HSCT	PD	Deceased

### 2.2 Patient case #2

16-year-old female presented with skin nodule in the left breast. Laboratory investigations revealed a WBC count of 7620 cells/microliter. She underwent excision, and histopathological examination showed monomorphic diffuse sheets of intermediate-sized neoplastic round cells that were positive for CD4, LCA, CD56 and CD123, consistent with BPDCN. There was no evidence of CNS or bone marrow involvement by the disease. Post excision, WB PET-CT scan revealed no other lesions elsewhere. The patient was initiated on the Hoelzer protocol (ALL-like regimen), and after 12 months while in maintenance therapy, her primary site skin disease progressed and no disease elsewhere. Patient received second-line hyper-CVAD therapy. After 3 cycles, she was in complete metabolic response and taken for haploidentical-HSCT with modified GIAC (G-CSF priming, intensified immunosuppression, ATG, and peripheral blood stem cell graft) conditioning regimen. Her transplant course was complicated by steroid-refractory acute GVHD of gut, transplant-associated TMA, late-onset haemorrhagic cystitis, CMV reactivation and chronic skin GVHD which improved in due course. She has been in complete response and on follow-up for past 2 years post-HSCT.

### 2.3 Patient case #3

40-year-old man presented with fatigue, fever, and generalized weakness for one month. Laboratory investigations revealed haemoglobin of 6 g/dL, a WBC count of 1,000 cells/ microliter, and platelets at 1,60,000 cells/microliter. BM flow cytometry showed positivity for CD45dim, CD117, CD4, CD56, CD113, HLA-DR, and CD38, consistent with BPDCN. Cytogenetic analysis revealed normal karyotype, while next-generation sequencing (NGS) identified an ASXL1 mutation. There was no CNS or other organ involvement. Started with A+V therapy and achieved CR after one cycle, though measurable residual disease (MRD) was detected by flow cytometry. He was then switched to ALL-like regimen (ICiCLe-ALL High protocol). He underwent allo-HSCT with a matched sibling donor (MSD) while in MRD-positive status. Conditioning regimen of cyclophosphamide and busulfan (CyBu) was used. Post-transplant, his clinical course was complicated by acute skin GVHD and veno-occlusive disease, both of which improved with treatment. However, he ultimately succumbed to lung complications due to bacterial pneumonia and overlap GVHD syndrome (skin and gastrointestinal). The patient remained MRD-positive post-transplant.

## 3 Discussion

Here, we present three cases of BPDCN from India, emphasizing its occurrence across all age groups and international locations, including one adolescent female. Previous data indicates that paediatric cases comprise approximately 10% and are typically considered less severe, with a higher likelihood of achieving long-term remission [10,11]. In this series, two patients presented with cutaneous involvement, another with BM involvement. Timely diagnosis and prompt initiation of effective frontline therapy are critical in managing rare diseases like BPDCN, where treatment options continue to evolve. Traditionally, chemotherapy was the mainstay of treatment but was associated with significant toxicity, particularly in elderly patients. The advent of novel therapy, TAG was approved for patients aged 2 years and older with newly diagnosed or relapsed/refractory BPDCN, marking a significant advancement in targeted therapy. Pemmaraju N et al. demonstrated the efficacy of TAG as upfront therapy. Among 65 patients, the ORR was 75%, with 57% achieving complete response (CR), and 51% successfully bridged to stem cell transplantation (SCT). The median overall survival (OS) was 38.4 months, and 72% remained in remission for ≥12 months post-SCT. In the relapsed setting (19 patients), TAG achieved an ORR of 58%, a previously unreported outcome [12]. Post-TAG era, five years after its approval, patients in our setting continue to be treated with standard upfront approaches, such as ALL-like regimens and A+V, due to financial constraints and high cost and limited availability of TAG.

Retrospective data from Feuillard et al., median overall survival (OS) following induction chemotherapy ranged from 12 -16 months. Total of 23 patients, 86% achieved CR, but 83% of those relapsed within a median of 9 months, highlighting the need for intensive consolidation therapy [13]. Reimer et al., found that patients treated with ALL-type regimens had better responses compared to those treated with AML-type or lymphoma-type therapies, but relapses were frequent [14]. Martin-Martin et al. treated 25 patients with intensive chemotherapy and observed better outcomes in those treated with ALL-type regimens, including lower relapse rates [15]. In our study, multiple induction chemotherapy regimens were used subsequently (Hoelzer protocol, ICiCLe-ALL protocol, hyperCVAD, A+V, CLV, VRD, miniCHOP, and Gemox) due to the lack of durable remission.

Consolidation with allo-HSCT is very important in the BPDCN management [16]. Two of our patients in this series underwent allo-HSCT (one haploidentical and one MSD). The EBMT reported outcomes of 34 patients who received allo-HCT, showing 3-year disease-free survival (DFS) of 33% and OS of 41% [17]. Kharfan-Dabaja et al. with 37 cases, reported 3-year OS of 58% [18]. A CIBTMR analysis by Murthy et al. showed a 5-year OS of 51.2% in patients with transplant. 3 years progression-free survival (PFS) was 85% and 55% at CR1 in patients with transplant and without transplant patients [19]. Early referral for allo-HSCT evaluation is essential, as it remains one of the most effective consolidative strategy in this aggressive malignancy [20].

## 4 Conclusion

Use of CD123-directed therapy is challenging due to financial and logistical barriers. Furthermore, CD123-directed agents are not yet widely available throughout the world [21]. Therefore, it is imperative that we as a field implement and report on non-CD123-directed regimens for treatment of patients with BPDCN [22]. Through our case scenarios, we highlighted the natural history, the therapeutic regimens employed, and the unique challenges faced in resource-limited settings [23,24]. While significant progress has been made in the understanding and management of BPDCN, further research and improved accessibility to novel therapies are crucial to enhance treatment outcomes [25]. Achieving complete responses that enable patients to proceed to allo-HSCT remains a key goal, underscoring the need for tailored strategies in our setting.

## Informed consent

Informed consent was obtained from each patient (or their legal representative/next of kin) for publication of clinical details and accompanying images in this case series of blastic plasmacytoid dendritic cell neoplasm (BPDCN). All identifying information has been omitted to ensure patient confidentiality.

## CRediT authorship contribution statement

**Paramasivam Sabitha:** Conceptualization, Data curation, Methodology, Writing – original draft, Writing – review & editing. **Aastha Goel:** Writing – original draft. **Santhosh Kumar:** Writing – original draft. **Chethan R:** Supervision, Writing – review & editing. **Sanal Fernandes:** Writing – review & editing. **Nitin Jain:** Supervision, Writing – review & editing. **Naveen Pemmaraju:** Supervision, Writing – review & editing. **Atul Sharma:** Supervision. **Ranjit Kumar Sahoo:** Supervision, Writing – review & editing.

## Declaration of competing interest

None.
